# MicroRNAs: Novel Players in Aortic Aneurysm

**DOI:** 10.1155/2015/831641

**Published:** 2015-06-28

**Authors:** Xian-ming Fu, Yang-zhao Zhou, Zhao Cheng, Xiao-bo Liao, Xin-min Zhou

**Affiliations:** ^1^Department of Cardiothoracic Surgery, The Second Xiangya Hospital, Central South University, Changsha, Hunan 410011, China; ^2^Department of Hematology, The Second Xiangya Hospital, Central South University, Changsha, Hunan 410011, China

## Abstract

An aortic aneurysm (AA) is a common disease with potentially life-threatening complications. Despite significant improvements in the diagnosis and treatment of AA, the associated morbidity and mortality remain high. MicroRNAs (miRNAs, miR) are small noncoding ribonucleic acids that negatively regulate gene expression at the posttranscriptional level by inhibiting mRNA translation or promoting mRNA degradation. miRNAs are recently reported to be critical modulators for vascular cell functions such as cell migration, contraction, differentiation, proliferation, and apoptosis. Increasing evidences suggest crucial roles of miRNAs in the pathogenesis and progression of cardiovascular diseases such as coronary artery disease, heart failure, arterial hypertension, and cardiac arrhythmias. Recently, some miRNAs, such as miR-24, miR-155, miR-205, miR-712, miR-21, miR-26a, miR-143/145, miR-29, and miR-195, have been demonstrated to be differentially expressed in the diseased aortic tissues and strongly associated with the development of AA. In the present paper, we reviewed the recent available literature regarding the role of miRNAs in the pathogenesis of AA. Moreover, we discuss the potential use of miRNAs as diagnostic and prognostic biomarkers and novel targets for development of effective therapeutic strategies for AA.

## 1. Introduction

An aortic aneurysm (AA) is defined as a localized or diffuse dilation of aorta with a diameter at least 1.5 times greater than the expected normal size [1]. Risk factors for AA development include aging, cigarette smoking, hypertension, family history, male gender, aging, and atherosclerosis (AS) [2, 3]. Despite improvements over the years in the diagnostic and therapeutic techniques for AA, the associated morbidity and mortality remain high. The most fatal clinical consequence of AA is acute rupture, which leads to a mortality as high as 90% in 2009 [4]. Current available treatments, such as prosthetic replacement (open surgery) or strengthening (endoprosthesis) of the aorta, are associated with a high mortality rate and limited durability, respectively [5]. Until now, no nonsurgical (medical) treatments have been approved for prevention or limitation of AA in humans. Not only is a better understanding of the molecular mechanisms of AA formation essential for understanding the physiological processes of this disease, but it is also important for identifying new biomarkers and therapeutic targets.

The mechanisms underlying AA are incompletely understood. AA formation is thought to be a multifactorial and predominantly degenerative process that results from a complex interplay between biological processes in the arterial wall and the hemodynamic stimuli on the wall [5–9]. The pathology of AA is characterized by endothelial dysfunction, chronic inflammation, vascular smooth muscle cell (VSMC) phenotype switch (earlier) and apoptosis (later), and extracellular matrix (ECM) degradation. Some unknown inciting events result in aortic wall injury, whereby inflammatory cells are recruited into the aortic wall. The infiltrated inflammatory cells such as macrophages and lymphocytes secrete various inflammatory cytokines and chemokines such as interleukin- (IL-) 1*β*, IL-6, tumor necrosis factor- (TNF-) *α*, and monocyte chemoattractant protein-1 (MCP-1). These cytokines and chemokines induce activation of matrix metalloproteinases (MMPs), particularly MMP-2 and MMP-9, and apoptosis of VSMC, which contribute prominently to AAs development [5]. Moreover, the catalytic activities of MMPs are partly controlled by specific inhibitors named tissue inhibitors of matrix metalloproteinases (TIMPs). TIMP1 binds to pro-MMP-9 forming a complex, while TIMP2 binds to pro-MMP-2. TIMPs were also found to play important roles in AA development [7].

MicroRNAs (miRNAs, miRs) are one class of small, conserved, single-stranded, noncoding RNA. They are approximately 18~25 nucleotides in length and can bind to complementary target sites in mRNA molecules, causing translation repression or the cleavage of the targets [10]. miRNAs have been reported to play important roles in a variety of pathophysiological processes [11–13]. Some recent studies have revealed that several miRNAs may be involved in vascular remodeling and pathogenesis of AA formation (Table 1). These findings clearly demonstrate the roles of miRNAs in the development of AA.

In the present paper, we reviewed the recent published literature about the role of miRNAs in the pathogenesis of AA. Moreover, we discussed their potential use as diagnostic and prognostic biomarkers and as novel targets for development of effective therapeutic strategies.

## 2. miRNAs in the Pathogenesis of AAs

### 2.1. miRNAs and Vascular Inflammation

Vascular inflammation contributes to the formation and development of AA [9]. Inflammatory reaction is characterized by infiltration of neutrophils, macrophages, T cells, and dendritic cells into the pathological aortic wall. Infiltrated inflammatory cells, particularly macrophages and lymphocytes, not only destruct directly ECM by releasing MMPs but also mediate activation of mesenchymal cells and apoptosis of VSMC, thus leading to progressive and pathological remodeling of aorta [14]. Recent studies have shown that miRNAs are involved in regulation of vascular inflammation and play crucial roles in AA development.

#### 2.1.1. miR-24

The highly conserved miR-23b-24-27b cluster is involved in postinfarct cardiac angiogenesis, cardiomyocyte survival, and cancer. AA progression is associated with downregulation of the miR-23b-24-27b cluster in murine abdominal AA models, with miR-24 displaying the most significant inverse regulation of its predicted targets in array profiling studies. Human abdominal AA also displays miR-24 downregulation, correlating inversely with aneurysm size. Maegdefessel group proved the function of miR-24 as a key regulator of vascular inflammation and abdominal AA pathology [15]. They revealed that chitinase 3-like 1 (CHI3L1) was a major target effector under the control of miR-24, regulating cytokine synthesis in macrophages as well as their survival, promoting aortic smooth muscle cell migration and cytokine production and stimulating adhesion molecule expression in vascular endothelial cells. Further, they showed that modulation of miR-24 alters abdominal AA progression in animal models and that miR-24 and CHI3L1 represent novel plasma biomarkers of abdominal AA disease progression in human.

#### 2.1.2. miR-126

miR-126 is a human microRNA that is highly expressed in endothelial cells (ECs) [16]. It is located within the 7th intron of the EGF-like domain 7 (EGFL7) gene, involved in cell migration and blood vessel formation. miR-126 is regulated by the binding of two transcription factors: ETS-1 and ETS-2 [17]. Recent studies showed that miR-126 is involved in the vascular integrity and angiogenesis. Knockout of miR-126 in mice and zebrafish has decreased vascular integrity and impaired proliferation, migration, and angiogenic activity of ECs [18]. It also has been reported to be involved in vascular inflammation by modulating vascular cell adhesion molecule- (VCAM-) 1 expression, which hence inhibits leukocyte adhesion and inflammation [19]. Additionally, miR-126 in ECs regulates vascular remodeling by modulating the expression of stromal cell-derived factor-1 (SDF-1) [20]. Furthermore, downregulation of miR-126 in plasma and upregulation in abdominal aortic aneurysmal tissues have been observed and indicate the potential role of miR-126 in AA formation [21]. These evidences showed that miR-126 regulates vascular inflammation and is involved in vascular remodeling and aortic diseases.

#### 2.1.3. miR-155

miR-155 is a typical multifunctional miRNA. It is expressed in a number of tissues and cell types and has been found to play a critical role in a wide variety of pathophysiological processes, including vascular inflammation. Chen and colleagues have reported that miR-155 was upregulated in primary murine macrophages and oxidized low density lipoprotein- (oxLDL-) stimulated monocytes, thus being involved in vascular inflammation [22]. In another study, Zhu and coworkers investigated the role of miR-155 in AS and found that miR-155 has increased in the aortic tissues of AS mice and in the plasma from patients with the coronary artery diseases [23]. They further showed that the miR-155 mimics have decreased secretion of IL-6 and TNF-*α* from oxLDL induced macrophages. On the contrary, the miR-155 inhibitor has promoted their secretions. Moreover, miR-155 has been shown to inhibit vascular inflammation and AS development by targeting mitogen-activated protein kinase 10 (MAP3K10). Taken together, all these evidences suggested that miR-155 represents an important modulator of vascular inflammation and may show important roles in inflammation associated vascular diseases, such as AS and AA.

#### 2.1.4. miR-181b

miR-181b is one member of the miR-181 family and has recently been shown to play an important role in vascular inflammation. It modulates vascular inflammation by targeting importin-*α*3 (IPOA3), a protein required for nuclear translocation of NF-*κ*B [24]. Sun and coworkers have showed that overexpression of miR-181b inhibited IPOA3 expression and downregulated NF-*κ*B-responsive gene (VCAM-1 and E-selectin) expression in ECs [25]. In an endotoxemic mice model, miR-181b expression decreased with proinflammatory stimuli, while it was rescued with miR-181b mimics administration. Moreover, decreased lung injury and mortality of mice were observed with miR-181b mimics treatment, which has been reported to be associated with reduced NF-*κ*B signaling and leukocyte influx in vascular endothelium. Furthermore, miR-181b expression in plasma was reduced in critically ill patients with sepsis. These findings indicated that miR-181b inhibits NF-*κ*B mediated inflammation through reducing the expression of IPOA3. In a sequent study [26], the same research group further investigated the role of miR-181b in the development of AS. miR-181b expression was reduced in the aortic intima and plasma from ApoE^−/−^ mice, as well as in the plasma from patients with coronary artery diseases. Systemic delivery of miR-181b resulted in overexpression of miR-181b and suppressed NF-*κ*B signaling and AS lesion formation. Collectively, these results demonstrated that miR-181b could inhibit vascular inflammation and AS development through suppressing NF-*κ*B activation. To date, there is no direct evidence about a role of miR-181b in the pathogenesis of AA; we believe that it may play a critical role because vascular inflammation is the central step of AA development.

#### 2.1.5. miR-223

Hemodynamic stress triggers vascular remodeling and infiltration of inflammatory cells, especially macrophages, into the intracranial aneurysmal walls. miR-223 is a hematopoietic specific microRNA with crucial functions in myeloid lineage development. It has also been reported to be a novel regulator of inflammation [27–29], which suppresses proinflammatory pathways and enhances the anti-inflammatory response. Overexpression of miR-223 shows a protective role for vascular homeostasis and inflammation [30]. Moreover, miR-223 was shown to be unregulated in abdominal AA tissues and negatively correlated with MCP-1, TNF-*α*, and TGF-*β* expression in diseased aortic tissues [21].

#### 2.1.6. miR-712 and miR-205

miR-712 is a murine specific atypical miRNA which derived from preribosomal RNA. miR-205 is thought to be potential human homolog of miR-712, which shares the same “seed sequence” and is highly conserved in most mammalian species including murine and human [31]. Recent studies have reported that microRNA-712 not only induces endothelial inflammation and AS but is also involved in AA formation. Son and colleagues have firstly identified that, by targeting 2 MMP inhibitors, tissue inhibitor of metalloproteinase 3 (TIMP3) and reversion-inducing cysteine-rich protein with Kazal motifs (RECK), miR-712 is an atypical mechanosensitive miRNA upregulated in ECs and suppresses endothelial inflammation and AS [31]. Furthermore, treatment with specific antagonist of miR-712 inhibited endothelial inflammation and AS in a TIMP3-dependent manner. They also found that human miR-205, homolog of miR-712, targets TIMP3 in a flow-dependent manner. In a sequent study from the same institute, they investigated the role of miR-712 and miR-205 in AA development [32]. miR-712 and miR-205 were shown to be underregulated in the abdominal aortic endothelium during angiotensionII (AngII) -induced AA in ApoE^−/−^ mice, associated with ECM degradation and AA development. Silencing of miR-712 and miR-205 by using anti-miR-712 and anti-miR-205 prevented AA development. Reduced aortic MMPs activity and vascular inflammation were observed. Moreover, upregulation of miR-205 expression was also identified in the human abdominal AA samples compared with the control. In summary, these results show that miR-712 and miR-205 stimulate MMPs activity and promote vascular inflammation by inhibiting TIMP3 and RECK, resulting in AA development. miR-712 and miR-205 may be potential modulators of AA.

### 2.2. miRNAs and VSMC Homeostasis

VSMC are the predominant cells in the media of aorta and essential in maintaining its structure and function through controlling proliferation and secretion and turnover of ECM. VSMC are plastic and can undergo reversible changes in their phenotypes in response to changing environmental cues. Two common phenotypic states of VSMC have been described: differentiated (also termed contractile) state and dedifferentiated (also termed synthetic) state. Differentiated phenotype is characterized by high levels of contractile gene expression and low rates of proliferation, migration, and ECM synthesis. Conversely, dedifferentiated phenotype has increased rates of proliferation, migration, and production of ECM, as well as reduced expression of contractile genes. In healthy vessels, VSMC can switch between states, but regulation of this switch is disrupted in vascular diseases and thought to contribute to the progression of diseases [33, 34]. Deregulation of phenotype switching and apoptosis of VSMC contribute to the development and progression of vascular pathologies like AA [35–38], and miRNAs are found to be critical modulators of VSMC function (phenotype) such as cell differentiation, contraction, migration, proliferation, calcification, and apoptosis [39–43]. Therefore, miRNAs are thought to be involved in AA formation. In several independent studies, miRNAs including miR-21, miR-26a, miR-126, miR-143/145, and miR-663 have been found to play crucial roles in AA development.

#### 2.2.1. miR-21

miR-21 is the first miRNA demonstrated to be involved in regulation of VSMC phenotype. It is highly expressed in VSMC and ECs and targets phosphatase and tensin homolog (PTEN) [44, 45], programmed cell death 4 (PDCD4) [46], sprouty-1 (SPRY1) [47], and B cell lymphoma 2 (BCL2) [45]. Ji group [45] found that miR-21 promotes VSMC proliferation and inhibits apoptosis by downregulating PTEN and upregulating BCL2. Davis group [48] has showed that miR-21 also promotes differentiation of VSMC in response to transforming growth factor-*β* (TGF-*β*) and bone morphogenetic protein (BMP) stimulation via a decrease in PDCD4 expression. These studies indicate that miR-21 is important in the maintenance of VSMC phenotype. More recently, Maegdefessel and colleagues have investigated the role of miR-21 in AA development [49]. They identified that miR-21 expression increased during AA formation. Overexpression of miR-21 inhibited AA expansion, which is associated with decreased apoptosis and downregulation of PTEN in the aortic wall. In contrast, systemic injection of a locked nucleic acid- (LNA-) modified antagomir targeting miR-21 led to a marked increase in the size of AA. Similar results were found in mice with AA augmented by nicotine and in patients with AA. Taken together, these data suggest that miR-21 is a key regulator of VSMC proliferation and apoptosis during AA development, and modulation of miR-21 expression may be a potential strategy to prevent AA formation.

#### 2.2.2. miR-26a

miR-26a is an important regulator of VSMC phenotype. miR-26a has been proved to inhibit VSMC differentiation and apoptosis and promote proliferation and migration through a mechanism that targets the TGF*β*/BMP pathway. Leeper and colleagues have performed a microarray-based study during the process of human aortic VSMC differentiation* in vitro* [50]. They identified that miR-26a is the highest-ranked significant differential expression of miRNA. VSMC differentiation was promoted by underexpression of miR-26a and inhibited by overexpression of miR-26a. In order to elucidate the mechanism that miR-26a modulates VSMC phenotype, the effects of miR-26a on the expression of the prodifferentiation TGF-*β*/BMP cascade molecules were assessed. Inhibition increased TGF-*β* superfamily signaling cascade gene expression including SMAD-1 and SMAD-4, while overexpression of miRNA-26a inhibited SMAD-1 expression. Furthermore, the expression of miR-26a in two murine AA models was evaluated and was found progressively downregulated during AA development. These results suggest that miR-26a may serve as an important regulator of VSMC biology and a potential therapeutic target in AA.

#### 2.2.3. miR-143/145

miR-143/145 cluster which is highly expressed in VSMC is the most studied miRNA cluster. miR-143 and miR-145 encoding genes are highly conserved and lie in close proximity with each other on murine chromosome 18 and human chromosome 5 [41, 42]. They have been shown to play crucial roles in regulating VSMC phenotypic switching and pathogenesis of vascular diseases. They modulate VSMC function through targeting several transcription factors, including Klf4, myocardin, and Elk-1. Two pioneering studies by Cheng group and Cordes group, respectively, investigated the role of miR-143/145 in determining VSMC phenotype* in vitro*. Cheng and colleagues have reported that overexpression of miR-145 promotes VSMC differentiation while miR-145 inhibitor represses differentiation [51]. In addition to regulating VSMC differentiation, miR-145 alone was reported to be able to maintain the differentiated spindle-like shape of VSMC and inhibit proliferation. The Cordes group [41] have further verified the regulatory effects of miR-145 on VSMC phenotype. The roles of miR-143/145 on VSMC phenotype have also been verified and validated* in vivo*. Recent studies have revealed the transition of VSMC from contractile phenotype to synthetic one in the media of thoracic aortic dissection (TAD) aorta [35]. Liao group [52] have reported that miR-143/145 were underexpressed in TAD, which may account for VSMC underdifferentiated in TAD and contributes to the aortic remodeling. In addition, a negative correlation between the expression of miR-143/145 and the dedifferentiation of VSMC has been observed. These results indicate that miR-143 and miR-145 are critical modulators of VSMC function and may play important roles in AA development.

#### 2.2.4. miR-663

miR-663 is highly expressed in ECs and VSMC. It is recently recognized as an important regulator of VSMC function. Li group have investigated the role of miR-663 in human VSMC phenotypic switch and the development of neointima formation [53]. They found that the expression of miR-663 decreased in human aortic VSMC with platelet-derived growth factor treatment but increased during VSMC differentiation. Furthermore, overexpression of miR-663 promotes VSMC differentiation and potently inhibits VSMC proliferation and migration, which are associated with downregulation of JunB and its downstream molecules, such as myosin light chain 9 (MLC9) and MMP-9. In addition, adeno-miR-663 suppressed the neointimal lesion formation after vascular injury via decreased JunB expression. Collectively, miR-663 is an important modulator of human VSMC phenotypic switch by targeting JunB/MLC9 expression and may represent an attractive approach for the treatment of AA.

### 2.3. miRNAs and Extracellular Matrix Remodeling

Aortic wall is comprised of layers of VSMC and an arrangement of ECM structural proteins, primarily collagen and elastin. AA is characterized by degradation of ECM, but the mechanisms underlying this process are incompletely understood. It has been established that TGF-*β* signaling plays key role in ECM remodeling and is involved in AA formation. TGF-*β*1 regulates the expression of certain miRNAs. In particularly, miR-29 and miR15 family have been increasingly noted to be associated with TGF-*β* signaling and ECM remodeling and AA development.

#### 2.3.1. miR-29

The miR-29 family including miR-29a, miR-29b, and miR-29c are enriched in fibroblasts and encoded by two separate loci. miR-29 family directly target at least 16 ECM genes such as collagen isoforms (COL1A1, COL1A2, and COL3A1), fibrillin-1 (FBN1), and elastin (ELN) and are involved in ECM remodeling in several organs [54, 55]. Moreover, it was recently indicated that miR-29 plays a pivotal role in the formation of aneurysm. Boon and colleagues [56] firstly discovered that the expression of miR-29 family increased in the aortic tissues of aged mice (18 months old) compared with young mice (6 weeks old), which is associated with a significant downregulation of ECM in aged mouse aortas. Furthermore, they found that systemic LNA-modified anti-miR-29 treatment decreased aortic dilatation in aged AngII treated mice, and this process is associated with increased expression of COL1A1, COL3A1, and ELN proteins. They also investigated miR-29 expression in human tissues from TAA patients and found that only miR-29b was upregulated among miR-29 family. In two murine AAA models (PPE and AngII infusion), Maegdefessel and colleagues [57] reported that miR-29b was the only member of miR-29 family decreased in aortic tissues during murine AAAs development. Anti-miR-29b treatment not only increased the expression of genes including Col1a1, Col2a1, Col3a1, Col5a1, and Eln, which encode type I, III, and V collagen and elastin, but also downregulated MMP-2 and MMP-9 expression. Accordingly, limited aneurysm expansion was observed with anti-miR-29b treatment. In contrast, overexpression of miR-29b led to rapid AAA expansion and increased aortic rupture rate. Merk and colleagues [58] further elucidated the role of miR-29b in early AAs development in murine model of Marfan syndrome (MFS). They found that the expression of miR-29b increased in ascending TAA of MFS mice, accompanied with increased apoptosis and MMP-2 activity as well as decreased expression of antiapoptotic proteins (Mcl-1 and Bcl-2) and elastin. Furthermore, an LNA-anti-miR-29b treatment limited AA development, aortic wall apoptosis, and ECM degradation. Taken together, these results provide important new insights into the mechanisms of AA formation and potentially allow for the development of new therapies.

#### 2.3.2. miR-195

Besides miR-29, Zampetaki group reported the miR-15 family to be the regulator of the collagen remodeling and the characteristic postnatal silencing of elastin [59]. Among the miR-15 family, miR-195 was proved to be differentially expressed in aortas of ApoE^−/−^ mice upon AngII infusion. Furthermore, the expression of miR-195 was altered in human aortic specimen with evidence of dissection. Direct binding of miR-195 to several ECM transcripts was detected in H4 cancer cells. Proteomic analysis of the secretome of murine aortic VSMC revealed that miR-195 targets a group of ECM proteins, including collagens, proteoglycans, elastin, and proteins associated with elastic microfibrils. In mice treated with antagomiR-195, higher aortic elastin expression was associated with an increase of MMP-2 and MMP-9. In human plasma, an inverse correlation of miR-195 was observed with the presence of abdominal AA and aortic diameter. Based on the evidences mentioned above, the miR-195 functioning as a potent regulator of the aortic ECM may contribute to the pathogenesis of AA disease. In addition, the plasma levels of miR-195 reduced in patients with AA suggested that it may serve as a noninvasive biomarker of AA.

## 3. Clinical Applications of MicroRNA in AA

### 3.1. The Role of miRNAs in the Diagnosis and Prognosis of AA

Not only are miRNAs tissue- and cell-specific but they also show different expression patterns. One miRNA may be highly expressed in one kind of cell or one tissue but has no or low expression in another kind of cells or tissues. Moreover, miRNAs are remarkably stable in the extracellular milieu, and they are detectable in blood and other body fluids. Circulating miRNAs have been demonstrated to share many of the essential characteristics of a good biomarker such as high degree of sensitivity and specificity, allowing early detection of pathological states; time-related changes during the course of disease; and a long half-life within the sample, as well as rapid and cost-effective laboratory detection. Accordingly, they have been shown to have roles in the diagnosis and prognosis of cardiovascular disease as biomarker. For example, miR-1, miR-133a, miR-499, and miR-208a have been reported to be upregulated in plasma of patients with acute MI, as a result of cardiomyocyte necrosis and massive release into the bloodstream [60]. The time dependent release of acute MI-related miRNAs has also been investigated: miR-1, miR-133a, and miR-208a increased continuously during the first 4 hours after the induction of MI, before conventional biomarkers of acute MI could be detected [61]. Furthermore, an inverse correlation of miR-195 in human plasma was observed with the presence of abdominal AA and aortic diameter. The plasma levels of miR-195 reduced in patients with abdominal AA suggested that it may serve as a noninvasive biomarker of abdominal AA [59]. These results indicate that circulating miRNAs could be used as biomarkers for diagnosis of MI in humans. More recently, the prognostic value of circulating miRNAs has been evaluated. miR-223 and miR-197 have been shown to have negative association, while miR-126 has a positive association with subsequent acute MI in a prospective study on a total of 19 miRNAs [62]. However, the potential role of miRNAs as biomarkers for the diagnosis and prognosis of aortic diseases has not yet been thoroughly evaluated. Several recent studies have identified differentially expressed miRNAs in aortic tissues as well as in plasma in aortic diseases [21, 63–65]. These miRNAs may not only contribute to the pathogenesis of aortic aneurysm or dissection but also provide biomarker potential. Further studies are necessary to explore the diagnostic and prognostic value of miRs as biomarkers in AA disease.

### 3.2. The Role of miRNAs in the Treatment of AA

As mentioned above, tissue- and cell-specific expression is one important characteristic of miRNA expression. Recent works have identified that changes in miRNA expression may contribute to the pathogenesis of AA. It is well established that multiple miRNAs are aberrantly expressed in diseased tissues. Several miRNAs-based treatments for AA have been proposed and reported in animal experiments according to the miRNA-based therapeutic strategies: restoring the expression of miRNAs reduced in diseases by miR-mimics or inhibiting overexpressed miRNAs by antagomirs (Table 2). The antagomirs (or anti-miRs) are small single-stranded antagonistic nucleotide sequences artificially synthesized to be perfectly complementary to a specific mature miRNA. When injected systemically or locally, antagomirs interact with miRNAs in the cytoplasm and hybridize specifically with the mature miRNA target hindering the binding of miRNA with their corresponding mRNA. Thus, antagomirs act as competitive inhibitors of miRNA and lead to a decrease in the effect caused by the excessive increase in the expression of certain miRNAs [66]. On the other hand, the miR-mimics are artificial small nucleotide sequences, double-stranded, similar to miRNA precursors (pre-miRNA). When introduced into the cells, the miR-mimics are recognized by the miRNA biogenesis machinery and processed by the enzyme dicer and subsequently incorporated into the RISC enzyme complex. Thus, the miR-mimics will function as a replacement of some miRNAs downregulated by setting the mRNA-target as endogenous miRNAs [67]. Additionally, there exist some other methods which can also inhibit miRNA expression and consequentially therapeutically interfere with the disease process such as masking, sponges, and erasers [68]. It should be noted that there are some challenges and risks for miRNA-based therapy like safety, delivery, and selectivity [68].

## 4. Summary

Our understanding of the molecular pathophysiology of AA is improving very rapidly but is still limited. miRNAs in AA have emerged as a new research area. The initial exciting results have demonstrated that multiple miRNAs are involved in the development of both human and animal AA diseases via regulating vascular cells differentiation, contraction, migration, proliferation, and apoptosis through their target genes. Additional studies with more patients and more animal models will be needed to determine the precise roles of miRNAs in AA. We believe that, on the basis of thorough understanding, miRNAs may represent novel promising biomarkers and new therapeutic targets for AA diseases.

## Figures and Tables

**Table 1:** MicroRNAs involved in vascular remodeling and aortic aneurysm (AA).

miRNA(s)	Cellular origin	Targets	Effects	References
miR-21	VSMC and EC	PTEN, SPRY1, PDCD4, and BCL2	Induces proliferation and decreases apoptosis of VSMC in AAA	[47, 49]

miR-24	VSMC and macrophage	CHI3L1	Inhibits vascular inflammation	[15]

miR-26a	VSMC	SMAD-1, SMAD-4	Promotes proliferation and inhibits differentiation, apoptosis of VSMC in AAA	[50]

miR-29	Fibroblast	COL1A1, COL1A2, COL3A1, FBN1, ELN	Downregulates ECM in AAA	[56–58, 69]

miR-126	EC	EGFL7	Inhibits vascular inflammation	[16, 18, 19]

miR-143/145	VSMC	Klf4, myocardin, Elk-1, and SRF	Promotes differentiation and represses proliferation of VSMC in AS and TAD	[41, 51, 52, 70]

miR-155	EC, BLC, TLC, macrophage, and DC	MAP3K10 CTLA4, SMAD2	Inhibits vascular inflammation in AS	[22, 23, 63]

miR-181b	EC	IPOA3	Inhibits vascular inflammation in AS	[24–26]

miR-195	VSMC	COL1A1, COL1A2, COL3A1, FBN1, ELN	Regulates MMPs in AAA	[59]

miR-223	Myeloid cell	CXCL2, CCL3, IL-6	Inhibits vascular inflammation in AAA	[21, 27–30]

miR-663	EC, VSMC	JunB, MLC9	Promotes differentiation and inhibits proliferation and migration of VSMC	[53, 71]

miR-712/miR-205	EC	TIMP3, RECK	Induces inflammation in AS and AAA	[31, 32, 72]

VSMC: vascular smooth muscle cell, EC: endothelial cell, BC: B lymphocytes, TC: T lymphocytes, DC: dendritic cells, AAA: abdominal aortic aneurysm, AS: atherosclerosis, TAD: thoracic aortic dissection, and ECM: extracellular matrix.

**Table 2:** Use of some miRNAs in the treatment of aortic aneurysm (AA).

miRNA	Model	Target	Anti-miR/mimics	Effects	Year/author
miR-21	Mice AngII-AAA PPE-AAA	PTEN	Mimics	Induces VSMC proliferation, decreases apoptosis via PTEN/PI3K/AKT, and prevents AA	2012 Maegdefessel et al. [49]

miR-24	Mice AngII-AAA PPE-AAA	CHI3L1	Mimics	Limits aortic vascular inflammation and AA formation	2014 Maegdefessel et al. [15]

miR-29	Aged mice AngII-AAA	Col1A1, Col3A1, elastin, Mcl-1	Antagomir, LNA-anti-miR	Induces ECM expression and inhibits AA formation	2011 Boon et al. [56]

miR-29b	Mice AngII-AAA PPE-AAA	Col1A1, Col3A1, elastin, Mcl-1	Antagomir, LNA-anti-miR	Increases collagen expression, leading to an early fibrotic response in the abdominal aortic wall, and prevents AAA progression	2012 Maegdefessel et al. [57]

miR-29b	Marfan mice Fbn1^C1039G/+^ AA	Col1A1, Col3A1, elastin, Mcl-1	Antagomir, LNA-anti-miR	Reduces ECM deposition and VSMC apoptosis and prevents AA formation	2012 Merk et al. [58]

miR-195	Mice AngII-AAA	Col1A1, Col3A1, elastin, Mcl-1	Antagomir, LNA-anti-miR	Reduces ECM deposition and VSMC apoptosis and prevents AA formation	2014 Zampetaki et al. [59]

miR-712/miR-205	Mice AngII-AAA	TIMP3, RECK	Antagomir, LNA-anti-miR	Inhibits both endothelial and circulating leukocyte inflammation Decreases MMPs activity and inflammation Prevents AAA formation	2014 Kim et al. [32]

AngII: angiotensin II, PPE: porcine pancreatic elastase, AAA: abdominal aortic aneurysm, LNA: alpha-linolenic acid, VSMC: vascular smooth muscle cell, ECM: extracellular matrix, and MMPs: matrix metalloproteinases.

## References

[1] JCS Joint Working Group (2013). Guidelines for diagnosis and treatment of aortic aneurysm and aortic dissection (JCS 2011): digest version. *Circulation Journal*.

[2] Singh K., Bønaa K. H., Jacobsen B. K., Bjørk L., Solberg S. (2001). Prevalence of and risk factors for abdominal aortic aneurysms in a population-based study: the Tromsø study. *American Journal of Epidemiology*.

[3] Wills A., Thompson M. M., Crowther M., Sayers R. D., Bell P. R. F. (1996). Pathogenesis of abdominal aortic aneurysms—cellular and biochemical mechanisms. *European Journal of Vascular and Endovascular Surgery*.

[4] Kochanek K. D., Xu J., Murphy S. L., Minino A. M., Kung H. C. (2009). Deaths: preliminary data for 2009. *National Vital Statistics Reports*.

[5] Golledge J., Muller J., Daugherty A., Norman P. (2006). Abdominal aortic aneurysm: pathogenesis and implications for management. *Arteriosclerosis, Thrombosis, and Vascular Biology*.

[6] Lu H., Rateri D. L., Bruemmer D., Cassis L. A., Daugherty A. (2012). Novel mechanisms of abdominal aortic aneurysms. *Current Atherosclerosis Reports*.

[7] Norman P. E., Davis T. M. E., Le M. T. Q., Golledge J. (2007). Matrix biology of abdominal aortic aneurysms in diabetes: mechanisms underlying the negative association. *Connective Tissue Research*.

[8] Thompson R. W., Geraghty P. J., Lee J. K. (2002). Abdominal aortic aneurysms: basic mechanisms and clinical implications. *Current Problems in Surgery*.

[9] Guo D.-C., Papke C. L., He R., Milewicz D. M. (2006). Pathogenesis of thoracic and abdominal aortic aneurysms. *Annals of the New York Academy of Sciences*.

[10] Li T., Cho W. C. S. (2012). MicroRNAs: mechanisms, functions and progress. *Genomics, Proteomics and Bioinformatics*.

[11] Carè A., Catalucci D., Felicetti F. (2007). MicroRNA-133 controls cardiac hypertrophy. *Nature Medicine*.

[12] Thum T., Galuppo P., Wolf C. (2007). MicroRNAs in the human heart: a clue to fetal gene reprogramming in heart failure. *Circulation*.

[13] van Rooij E., Sutherland L. B., Liu N. (2006). A signature pattern of stress-responsive microRNAs that can evoke cardiac hypertrophy and heart failure. *Proceedings of the National Academy of Sciences of the United States of America*.

[14] Pearce W. H., Koch A. E. (1996). Cellular components and features of immune response in abdominal aortic aneurysms. *Annals of the New York Academy of Sciences*.

[15] Maegdefessel L., Spin J. M., Raaz U. (2014). miR-24 limits aortic vascular inflammation and murine abdominal aneurysm development. *Nature Communications*.

[16] Wang S., Aurora A. B., Johnson B. A. (2008). The endothelial-specific microRNA miR-126 governs vascular integrity and angiogenesis. *Developmental Cell*.

[17] Harris T. A., Yamakuchi M., Kondo M., Oettgen P., Lowenstein C. J. (2010). Ets-1 and Ets-2 regulate the expression of MicroRNA-126 in endothelial cells. *Arteriosclerosis, Thrombosis, and Vascular Biology*.

[18] Fish J. E., Santoro M. M., Morton S. U. (2008). miR-126 regulates angiogenic signaling and vascular integrity. *Developmental Cell*.

[19] Harris T. A., Yamakuchi M., Ferlito M., Mendell J. T., Lowenstein C. J. (2008). MicroRNA-126 regulates endothelial expression of vascular cell adhesion molecule 1. *Proceedings of the National Academy of Sciences of the United States of America*.

[20] van Solingen C., de Boer H. C., Bijkerk R. (2011). MicroRNA-126 modulates endothelial SDF-1 expression and mobilization of Sca-1^+^/Lin^−^ progenitor cells in ischaemia. *Cardiovascular Research*.

[21] Kin K., Miyagawa S., Fukushima S. (2012). Tissue- and plasma-specific MicroRNA signatures for atherosclerotic abdominal aortic aneurysm. *Journal of the American Heart Association*.

[22] Chen T., Huang Z., Wang L. (2009). MicroRNA-125a-5p partly regulates the inflammatory response, lipid uptake, and ORP9 expression in oxLDL-stimulated monocyte/macrophages. *Cardiovascular Research*.

[23] Zhu J., Chen T., Yang L. (2012). Regulation of microRNA-155 in atherosclerotic inflammatory responses by targeting MAP3K10. *PLoS ONE*.

[24] Sun X., Sit A., Feinberg M. W. (2014). Role of miR-181 family in regulating vascular inflammation and immunity. *Trends in Cardiovascular Medicine*.

[25] Sun X., Icli B., Wara A. K. (2012). MicroRNA-181b regulates NF-*κ*B-mediated vascular inflammation. *The Journal of Clinical Investigation*.

[26] Sun X., He S., Wara A. K. M. (2014). Systemic delivery of MicroRNA-181b inhibits nuclear factor-kappaB activation, vascular inflammation, and atherosclerosis in apolipoprotein E-deficient mice. *Circulation Research*.

[27] Johnnidis J. B., Harris M. H., Wheeler R. T. (2008). Regulation of progenitor cell proliferation and granulocyte function by microRNA-223. *Nature*.

[28] Zhuang G., Meng C., Guo X. (2012). A novel regulator of macrophage activation: miR-223 in obesity-associated adipose tissue inflammation. *Circulation*.

[29] Lee H.-J., Yi J.-S., Lee H.-J., Lee I.-W., Park K.-C., Yang J.-H. (2013). Dysregulated expression profiles of microRNAs of experimentally induced cerebral aneurysms in rats. *Journal of Korean Neurosurgical Society*.

[30] Kanematsu Y., Kanematsu M., Kurihara C. (2011). Critical roles of macrophages in the formation of intracranial aneurysm. *Stroke*.

[31] Son D. J., Kumar S., Takabe W. (2013). The atypical mechanosensitive microRNA-712 derived from pre-ribosomal RNA induces endothelial inflammation and atherosclerosis. *Nature Communications*.

[32] Kim C. W., Kumar S., Son D. J., Jang I. H., Griendling K. K., Jo H. (2014). Prevention of abdominal aortic aneurysm by anti-microRNA-712 or anti-microRNA-205 in angiotensin II-infused mice. *Arteriosclerosis, Thrombosis, and Vascular Biology*.

[33] Rensen S. S. M., Doevendans P. A. F. M., Van Eys G. J. J. M. (2007). Regulation and characteristics of vascular smooth muscle cell phenotypic diversity. *Netherlands Heart Journal*.

[34] Owens G. K., Kumar M. S., Wamhoff B. R. (2004). Molecular regulation of vascular smooth muscle cell differentiation in development and disease. *Physiological Reviews*.

[35] Lesauskaite V., Tanganelli P., Sassi C. (2001). Smooth muscle cells of the media in the dilatative pathology of ascending thoracic aorta: morphology, immunoreactivity for osteopontin, matrix metalloproteinases, and their inhibitors. *Human Pathology*.

[36] Henderson E. L., Geng Y.-J., Sukhova G. K., Whittemore A. D., Knox J., Libby P. (1999). Death of smooth muscle cells and expression of mediators of apoptosis by T lymphocytes in human abdominal aortic aneurysms. *Circulation*.

[37] Riches K., Angelini T. G., Mudhar G. S. (2013). Exploring smooth muscle phenotype and function in a bioreactor model of abdominal aortic aneurysm. *Journal of Translational Medicine*.

[38] Ailawadi G., Moehle C. W., Pei H. (2009). Smooth muscle phenotypic modulation is an early event in aortic aneurysms. *Journal of Thoracic and Cardiovascular Surgery*.

[39] Liao X.-B., Zhang Z.-Y., Yuan K. (2013). MiR-133a modulates osteogenic differentiation of vascular smooth muscle cells. *Endocrinology*.

[40] Cui R.-R., Li S.-J., Liu L.-J. (2012). MicroRNA-204 regulates vascular smooth muscle cell calcification in vitro and in vivo. *Cardiovascular Research*.

[41] Cordes K. R., Sheehy N. T., White M. P. (2009). miR-145 and miR-143 regulate smooth muscle cell fate and plasticity. *Nature*.

[42] Boettger T., Beetz N., Kostin S. (2009). Acquisition of the contractile phenotype by murine arterial smooth muscle cells depends on the *Mir143/145* gene cluster. *The Journal of Clinical Investigation*.

[43] Davis-Dusenbery B. N., Wu C., Hata A. (2011). Micromanaging vascular smooth muscle cell differentiation and phenotypic modulation. *Arteriosclerosis, Thrombosis, and Vascular Biology*.

[44] Roy S., Khanna S., Hussain S.-R. A. (2009). MicroRNA expression in response to murine myocardial infarction: MiR-21 regulates fibroblast metalloprotease-2 via phosphatase and tensin homologue. *Cardiovascular Research*.

[45] Ji R., Cheng Y., Yue J. (2007). MicroRNA expression signature and antisense-mediated depletion reveal an essential role of MicroRNA in vascular neointimal lesion formation. *Circulation Research*.

[46] Liu X., Cheng Y., Yang J., Krall T. J., Huo Y., Zhang C. (2010). An essential role of PDCD4 in vascular smooth muscle cell apoptosis and proliferation: implications for vascular disease. *American Journal of Physiology—Cell Physiology*.

[47] Thum T., Gross C., Fiedler J. (2008). MicroRNA-21 contributes to myocardial disease by stimulating MAP kinase signalling in fibroblasts. *Nature*.

[48] Davis B. N., Hilyard A. C., Lagna G., Hata A. (2008). SMAD proteins control DROSHA-mediated microRNA maturation. *Nature*.

[49] Maegdefessel L., Azuma J., Toh R. (2012). MicroRNA-21 blocks abdominal aortic aneurysm development and nicotine-augmented expansion. *Science Translational Medicine*.

[50] Leeper N. J., Raiesdana A., Kojima Y. (2011). MicroRNA-26a is a novel regulator of vascular smooth muscle cell function. *Journal of Cellular Physiology*.

[51] Cheng Y., Liu X., Yang J. (2009). MicroRNA-145, a novel smooth muscle cell phenotypic marker and modulator, controls vascular neointimal lesion formation. *Circulation Research*.

[52] Liao M., Zou S., Weng J. (2011). A microRNA profile comparison between thoracic aortic dissection and normal thoracic aorta indicates the potential role of microRNAs in contributing to thoracic aortic dissection pathogenesis. *Journal of Vascular Surgery*.

[53] Li P., Zhu N., Yi B. (2013). MicroRNA-663 regulates human vascular smooth muscle cell phenotypic switch and vascular neointimal formation. *Circulation Research*.

[54] van Rooij E., Sutherland L. B., Thatcher J. E. (2008). Dysregulation of microRNAs after myocardial infarction reveals a role of miR-29 in cardiac fibrosis. *Proceedings of the National Academy of Sciences of the United States of America*.

[55] Ott C. E., Grünhagen J., Jäger M. (2011). MicroRNAs differentially expressed in postnatal aortic development downregulate elastin via 39′ UTR and coding-sequence binding sites. *PLoS ONE*.

[56] Boon R. A., Seeger T., Heydt S. (2011). MicroRNA-29 in aortic dilation: implications for aneurysm formation. *Circulation Research*.

[57] Maegdefessel L., Azuma J., Toh R. (2012). Inhibition of microRNA-29b reduces murine abdominal aortic aneurysm development. *The Journal of Clinical Investigation*.

[58] Merk D. R., Chin J. T., Dake B. A. (2012). MiR-29b participates in early aneurysm development in Marfan syndrome. *Circulation Research*.

[59] Zampetaki A., Attia R., Mayr U. (2014). Role of miR-195 in aortic aneurysmal disease. *Circulation Research*.

[60] Condorelli G., Latronico M. V. G., Cavarretta E. (2014). MicroRNAs in cardiovascular diseases: current knowledge and the road ahead. *Journal of the American College of Cardiology*.

[61] Liebetrau C., Möllmann H., Dörr O. (2013). Release kinetics of circulating muscle-enriched microRNAs in patients undergoing transcoronary ablation of septal hypertrophy. *Journal of the American College of Cardiology*.

[62] Zampetaki A., Willeit P., Tilling L. (2012). Prospective study on circulating microRNAs and risk of myocardial infarction. *Journal of the American College of Cardiology*.

[63] Biros E., Moran C. S., Wang Y., Walker P. J., Cardinal J., Golledge J. (2014). microRNA profiling in patients with abdominal aortic aneurysms: the significance of miR-155. *Clinical Science*.

[64] Liu G., Huang Y., Lu X. (2010). Identification and characteristics of microRNAs with altered expression patterns in a rat model of abdominal aortic aneurysms. *Tohoku Journal of Experimental Medicine*.

[65] Pahl M. C., Derr K., Gäbel G. (2012). MicroRNA expression signature in human abdominal aortic aneurysms. *BMC Medical Genomics*.

[66] Krützfeldt J., Rajewsky N., Braich R. (2005). Silencing of microRNAs in vivo with ‘antagomirs’. *Nature*.

[67] van Rooij E., Marshall W. S., Olson E. N. (2008). Toward microRNA-based therapeutics for heart disease: the sense in antisense. *Circulation Research*.

[68] Caroli A., Cardillo M. T., Galea R., Biasucci L. M. (2013). Potential therapeutic role of microRNAs in ischemic heart disease. *Journal of Cardiology*.

[69] Maegdefessel L., Azuma J., Tsao P. S. (2014). MicroRNA-29b regulation of abdominal aortic aneurysm development. *Trends in Cardiovascular Medicine*.

[70] Elia L., Quintavalle M., Zhang J. (2009). The knockout of miR-143 and -145 alters smooth muscle cell maintenance and vascular homeostasis in mice: correlates with human disease. *Cell Death and Differentiation*.

[71] Ni C.-W., Qiu H., Jo H. (2011). MicroRNA-663 upregulated by oscillatory shear stress plays a role in inflammatory response of endothelial cells. *American Journal of Physiology: Heart and Circulatory Physiology*.

[72] Maegdefessel L., Spin J. M., Tsao P. S. (2014). New ways to dismantle a ticking time bomb: microRNA 712/205 and abdominal aortic aneurysm development. *Arteriosclerosis, Thrombosis, and Vascular Biology*.

